# Comparative Analysis of Nutritional, Textural, and Sensory Attributes of Butter Crab and Normal Female Mud Crab (*Scylla paramamosain*): Insights for Market Positioning and Consumer Preference

**DOI:** 10.3390/foods14122101

**Published:** 2025-06-15

**Authors:** Baojia Chen, Mingfei Feng, Kang Fang, Kun Wu, Hai Yang, Shaojian Chen, Haoji Guo, Shuangli Hao, Xiaobo Wen

**Affiliations:** 1 Nansha-South China Agricultural University Fishery Research Institute Guangdong, Guangzhou 511464, China; baojiachen2025@163.com (B.C.); fengmingfei2024@163.com (M.F.); fangkang@stu.scau.edu.cn (K.F.); wk@scau.edu.cn (K.W.); 18270114173@163.com (H.Y.); 13922334904@139.com (S.C.); haojiguostu@163.com (H.G.); 2 College of Marine Sciences, South China Agricultural University, Guangzhou 510642, China

**Keywords:** butter crab, normal female mud crab, nutritional profiles, texture analysis, sensory characteristics

## Abstract

In recent years, the butter crab (BC), a distinctive phenotypic variant of the female mud crab *Scylla paramamosain*, has garnered increasing market attention due to its perceived superior nutritional and sensory attributes. This study conducted a comprehensive comparative analysis of the nutritional composition, textural properties, and sensory characteristics of BC and normal female mud crab (NFMC). Results showed that the muscle and hepatopancreas of BC contained significantly higher lipid contents (0.77 and 22.14 g/100 g wet weight) and elevated levels of DHA + EPA (18.36% and 12.86% of total fatty acids), which contributed to its characteristic orange–yellow coloration, as reflected by colorimetric values (L* × a* × b* = 30.20 × 4.38 × 16.15). Sensory evaluation revealed that BC exhibited enhanced umami taste and aroma in both muscle and hepatopancreas, corresponding to higher concentrations of umami amino acids (0.75 and 1.95 mg/g wet weight) and aldehydes (35.06% and 34.37% of total volatiles), respectively. In addition, 80% of panelists preferred BC based on visual appearance, indicating its strong consumer appeal. Overall, this study advances our understanding of the biochemical and sensory profiles of BC and NFMC and provides important insights for market positioning of BC in the premium seafood sector.

## 1. Introduction

The mud crab (*Scylla paramamosain*) is a marine crustacean of high economic value, widely distributed across the Indo-West Pacific region and along the southern coastal areas of China [1,2,3]. Renowned for its rapid growth, large size, and strong market demand, this species has become a key aquaculture commodity. In China alone, mud crab production reached 224,091 tons in 2023, with aquaculture accounting for 70.1% (157,012 tons) of the total yield [4]. However, market demand continues to outpace supply, underscoring the need for innovations in mud crab farming and product diversification.

In recent years, a unique variety of female mud crab known locally as the “butter crab” (BC) or “yellow oil crab” has emerged in Guangdong Province, China [5,6,7,8]. Characterized by its yellow–orange, lipid-rich hemolymph and underdeveloped ovaries, BC displays a distinct phenotype compared to normal female mud crabs (NFMC). This phenotype is believed to result from environmental stressors such as elevated temperatures and low salinity [6,8], leading to the accumulation of lipids and carotenoids throughout the body. Due to its rarity—estimated at one in several thousand—BC commands a premium price in the market, often exceeding 300 dollars per specimen [9]. It is celebrated not only for its scarcity but also for its exceptional sensory qualities and nutritional richness.

Despite increasing commercial interest, the scientific understanding of BC remains limited. Previous studies have primarily focused on its basic biochemical and nutritional attributes [5,6,7], while other critical factors influencing consumer preference—such as texture, appearance, aroma, and taste—have been largely overlooked [10,11]. Furthermore, no integrated study has systematically compared the sensory and nutritional profiles of BC and NFMC using advanced analytical and sensory evaluation technologies.

To address this gap, the present study represents the first comprehensive investigation of both the nutritional value and sensory characteristics of BC in comparison to NFMC. We assessed three key edible tissues—muscle, hepatopancreas, and ovaries—using a multidisciplinary approach. Chemical composition analysis included proximate nutrients, free amino acids, nucleotides, and fatty acids. Sensory attributes were objectively evaluated using instrumental colorimetry, texture analysis, electronic nose (E-nose), and electronic tongue (E-tongue) technologies. Additionally, volatile flavor compounds were profiled via headspace solid-phase microextraction coupled with gas chromatography–mass spectrometry (HS-SPME-GC/MS).

This integrated analysis provides novel insights into the distinctive qualities of butter crab and lays the groundwork for its differentiation in high-end seafood markets. Importantly, it also supports the development of targeted aquaculture strategies to enhance the sustainable production of this high-value phenotype. By combining nutritional and sensory data, our study offers a holistic evaluation that bridges consumer preferences with aquaculture innovation, contributing meaningfully to both scientific knowledge and industry practice.

## 2. Materials and Methods

### 2.1. Sample Preparation

Female *S. paramamosain*, in two growth forms (NFMC and BC, with body weights of 302.22 ± 6.35 g and 267.71 ± 5.16 g, respectively), were supplied by Guangzhou Butter Crab Aquatic Products Co., Ltd. (Guangzhou, China). Furthermore, 23 individuals, each of actively intact NFMC and BC, were randomly selected and sampled. The crabs were anesthetized in ice for 15 min, after which surface moisture was wiped off with a paper towel. The crabs were subsequently weighed using an electronic balance (AB1202, Shinko Ltd., Tokyo, Japan). The carapace of each crab was dissected using scissors, and the ovary, hepatopancreas, and all muscle tissues from the carapace, chelae, walking legs, and swimming legs were collected and weighed. The muscle yield, hepatopancreas index, gonadosomatic index, and total edible yield were calculated according to the equations in reference [12]. Additionally, muscle tissue from the large chelipeds, along with tissues from the hepatopancreas and ovary, were collected for further analysis. All procedures were conducted in accordance with the ethical guidelines approved by the Institutional Animal Care and Use Committee of the Nansha-South China Agricultural University Fishery Research Institute, Guangzhou, China.

### 2.2. Proximate Chemical Composition Determination

The moisture contents of the muscle, hepatopancreas, and ovaries of NFMC and BC were measured by drying the sample at 110 °C until a constant weight was achieved. Crude protein content of samples (muscle, hepatopancreas, and ovaries) was assessed using the Kjeldahl method, with a nitrogen-to-protein conversion factor of 6.25. The total lipid content of samples (muscle, hepatopancreas, and ovaries) was determined through Soxhlet extraction, while the ash content was obtained by combusting the samples at 550 °C. All quantification techniques followed the methods as described previously [1].

### 2.3. Quantitative Color Measurement

Color measurements were quantitatively taken at the arthrodial membrane on the surface of the swimming leg using a colorimeter (NR110, 3nh Ltd., Shenzhen, China) based on the CIELAB color system. The color parameters recorded included L* (where L* = 0 corresponds to black and L* = 100 corresponds to white), a* (representing red (+) and green (−)), and b* (indicating yellow (+) and blue (−)). Measurements were repeated three times at the same location to calculate the average values and standard deviations for each color parameter.

### 2.4. Visual Sensory Evaluation After Cooking

The NFMC and BC, which had been cleaned and anesthetized with ice, were placed in a food steamer. After steaming for 15 min, the cooked NFMC and BC were placed on a clean white porcelain plate and then cut in half along the centerline of the carapace for visual sensory evaluation.

A total of 30 trained panelists (15 males and 15 females, aged 22–35), all with prior experience in seafood sensory evaluation, participated in the visual assessment. The evaluation was conducted in individual sensory booths under standardized white lighting to ensure consistent visual conditions and to prevent interaction among participants. Each sample was anonymized using a random three-digit code and presented in a randomized order to eliminate bias. Panelists were instructed to evaluate each sample using a 10-point hedonic scale (1 = extremely poor, 10 = extremely good), based on the following visual attributes: external appearance (shell color, glossiness, and structural integrity), muscle and hepatopancreas appearance (color, oiliness, and firmness), and overall visual appeal. In addition to numerical scoring, panelists were also asked to indicate their preferred sample based solely on visual appearance.

### 2.5. Texture Analysis

Muscle tissues from the large chelipeds, hepatopancreas, and ovary were collected and steamed for 10 min prior to texture profile analysis (TPA). The texture profiles of these tissues were measured using a TA-XT Plus analyzer (Stable Micro Systems, Godalming, UK), following the method as previously described [13], with some modifications. The double compression TPA mode was employed with the following settings: a test speed of 2.0 mm/s, compression deformation of 50%, and a trigger force of 5 g. Each sample, with dimensions of 1.5 × 1.5 × 0.5 cm, was measured at room temperature, with each test repeated three times. The following texture parameters were calculated based on the force–time curve obtained during analysis: hardness, brittleness, springiness, cohesiveness, gumminess, chewiness, and resilience.

### 2.6. Fatty Acid Analysis

Fatty acid content of the muscle, hepatopancreas, and ovaries of NFMC and BC was determined using the method outlined in our previous study [1]. Total lipids from the hepatopancreas, ovary, and muscle were extracted after homogenization in a chloroform–methanol mixture (2:1, *v*/*v*), following the protocol as previously described [14]. The tissue lipids were then dissolved in 2 mL of a 2% (*w*/*v*) sodium hydroxide methanol solution, derivatized with 2 mL of boron trichloride–methanol (14%, *w*/*v*), and heated in a water bath at 60 °C for 15 min. After cooling to room temperature, 2 mL of n-hexane was added, and the mixture was gently shaken to extract the fatty acid methyl esters (FAME). Anhydrous sodium sulfate was used to remove any moisture from the n-hexane extract. FAMEs were analyzed using gas chromatography (7890B, Agilent Ltd., Santa Clara, CA, USA). The establishment of the specified parameters was outlined in our earlier study [1]. The fatty acid profiles were determined by comparing the retention times of the Supelco 37 Component FAME Mix (Sigma-Aldrich Ltd., St. Louis, MO, USA). The fatty acid content in each sample was represented as g/100 g total fatty acids (g/100 g).

### 2.7. Free Amino Acid Analysis

The free amino acid content of the muscle, hepatopancreas, and ovaries of NFMC and BC was analyzed using a method as described previously with minor modifications [6]. In this procedure, 1 g of freeze-dried sample was ground, and 10 mL of 4% sulfosalicylic acid was added. The mixture was stirred for 1 h to ensure complete extraction. The resulting solution was then centrifuged at 12,000 rpm for 30 min at 4 °C. Afterward, 1 mL of the supernatant was filtered through a 0.22 μm aqueous-phase filter membrane, and the filtrate was analyzed using a Hitachi 835-50 automatic amino acid analyzer (Hitachi Ltd., Tokyo, Japan).

### 2.8. Free Nucleotide Assays

High-performance liquid chromatography (HPLC) (AG1100, Agilent Ltd., Santa Clara, CA, USA) was employed to quantify free nucleotides in tissue samples, using external standards as described previously [15]. To prepare the samples, 1 g of tissue was weighed, rapidly frozen in liquid nitrogen, and then ground. The ground tissue was transferred to a centrifuge tube containing 1 mL of 5% trichloroacetic acid. The samples were then centrifuged at 12,000 rpm for 15 min. After centrifugation, the supernatants were collected, filtered through a 0.22 μm filter, and analyzed using HPLC.

### 2.9. Equivalent Umami Concentration

The equivalent umami concentration (EUC) method as developed by previous reference was used to assess the umami intensity [16]. EUC measures the umami intensity of a mixture of umami amino acids and flavor nucleotides in relation to monosodium glutamate (MSG). The umami concentration is quantified based on the following equation [16], which allows for comparison of the sample’s umami level with that of MSG.

### 2.10. E-Nose Analysis

E-nose analysis was conducted using a PEN 3.0 E-nose device (Winmuster Airsense Analytics Inc., Schwerin, Germany), which is equipped with a sensor array consisting of ten metal-oxide semiconductors with varying chemical compositions and thicknesses. The sensors used were W1C, W5S, W3C, W6S, W5C, W1S, W1W, W2S, W2W, and W3S, designed to detect a range of compounds including aromatic compounds, oxynitride, ammonia, hydrogen, alkanes, methane, sulfur compounds, ethanol, aromatic and organic sulfur compounds, and alkanes. In the procedure, a 1.3 g sample (the muscle, hepatopancreas, and ovaries of NFMC and BC) was added to a 20 mL headspace vial, sealed with a PTFE septum, and allowed to equilibrate at 60 °C for 10 min prior to analysis. The headspace gas was then injected into the E-nose via a constant flow of air at a rate of 400 mL/min for 60 s, while sensor signals were recorded every second during the test. After each analysis, the sensor system was flushed with filtered air for 120 s to reset the baseline before the next sample was tested. Data were gathered using pattern recognition software (WinMuster, v.1.6, Airsense Analytics GmbH., Schwerin, Germany). Three samples in each group were analyzed, and the average was used for subsequent statistical evaluation.

### 2.11. E-Tongue Analysis

The taste profile of the muscle, hepatopancreas, and ovaries of NFMC and BC was analyzed using the electronic tongue system (SA-402B, Insent Inc., Atsugi, Japan). This system includes nine taste sensors designed to assess various taste attributes, such as sweetness, bitterness, sourness, umami, saltiness, aftertaste-A, aftertaste-B, richness, and astringency. Prior to testing, all sensors were conditioned for 24 h with an internal solution (3.3 M KCl and saturated silver chloride) and a reference solution (30 mM KCl and 0.3 mM tartaric acid). After being steamed for 10 min, the sample was homogenized and mixed with water in a 1:5 ratio (30 g of sample to 150 mL of water). The mixture was centrifuged at 3000 rpm for 10 min. After centrifugation, 105 mL of the supernatant was collected for the measurement of the nine taste values. Three samples in each group were analyzed, and the averages were used for subsequent statistical evaluation.

### 2.12. Volatile Compound Analysis

The volatile compounds from the muscle, hepatopancreas, and ovaries of NFMC and BC were analyzed using HS-SPME-GC/MS. The samples in glass vials were heated to 90 °C for 60 min, allowing volatile compounds to be absorbed by a monotrap (RCC 18, GL Science Ltd., Tokyo, Japan) in the headspace. The compounds concentrated in the monotrap were subsequently desorbed in a thermal desorption unit (TDU, Gerstel GmbH & Co., KG, Mülheim an der Ruhr, Germany) and injected into a GC–MS system (6890A-5975C, Agilent, Santa Clara, CA, USA) equipped with a cooled injection system (CIS, Gerstel). The GC–MS parameters were as follows: the column used was DB-5MS (60 m × 0.32 mm; Agilent); high-purity helium (99.999%) was used as the carrier gas at a flow rate of 1.2 mL/min; the detector interface temperature was set to 250 °C; the ion source temperature was 230 °C; and the ionization energy was 70 eV. The oven temperature was programmed to increase from 40 °C to 100 °C at a rate of 5 °C/min, then to 180 °C at 3 °C/min, and finally to 240 °C at 5 °C/min, where it was held for 5 min. Identification of volatile compounds was achieved through NIST library search (NIST 08) and retention index (RI) matching.

### 2.13. Statistical Analysis

The data were analyzed utilizing SPSS software (version 27, SPSS Inc., Chicago, IL, USA), with results reported as the means ± standard deviations (SD). For normally distributed data with equal variances between groups, comparisons of means were conducted using Student’s *t*-test (independent samples). When data did not meet the assumptions of normality, differences in medians between groups were assessed using the Wilcoxon–Mann–Whitney U test (rank-sum test). Statistical significance was set at *p* < 0.05. Multivariate analyses, including 3D scatter plots of color parameter data, principal components analysis (PCA) of E-nose/tongue data, radar maps of E-nose/tongue data, and heatmap analysis of fatty acid results, were performed using Origin 2023 software (Origin Lab., Northampton, MA, USA). The bar chart was performed using GraphPad Prism Software (version 10, GraphPad Software Inc., San Diego, CA, USA). All assays were conducted in triplicate or more.

## 3. Results and Discussion

### 3.1. Visual Sensory Evaluation

**Figure 1:**
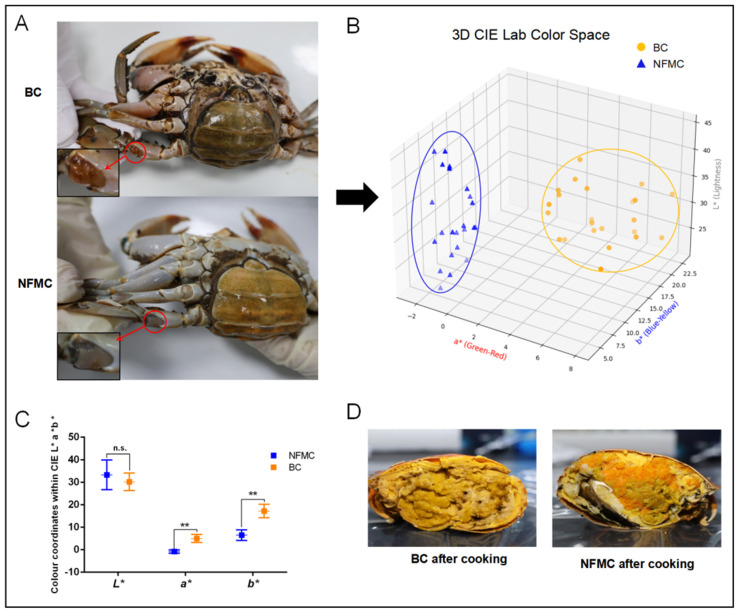
The differences in appearance phenotype and cross-sectional diagram of NFMC and BC. (**A**) in the arthrodial membrane (arrow) of the legs of NFMC and BC; 3D scatter plot (**B**) and grouped scatter plot with lines (**C**) of color parameters in the arthrodial membrane of the legs of NFMC and BC; cross-sectional diagram (**D**) of NFMC and BC after cooking. Data are expressed as mean ± standard deviation (*n* = 23). NFMC, normal female mud crab; BC, butter crab; n.s., no significant difference; ** mean *p* < 0.01, respectively.

The appearance of BC differs significantly from that of NFMC. The arthrodial membrane of the swimming legs in BC exhibits a distinctive orange–yellow color, while the NFMC shows a more typical off-white hue (Figure 1A). A more detailed analysis of color parameters, as shown in Figure 1B, reveals that the BC group is clearly distinguishable from the NFMC group in the 3D score plot. Additionally, Figure 1C highlights a significant difference in the a* and b* values between the two groups (*p* < 0.01), with the BC group exhibiting markedly higher a* (4.38 ± 2.36) and b* (16.15 ± 3.34) values. These increases in a* and b* values are indicative of a reddening and yellowing of the arthrodial membrane in BC, likely due to the deposition of pigmentation, which plays a critical role in determining the color of both fish and shellfish [17]. These findings suggest that the unique color of the BC arthrodial membrane is likely a result of higher levels of lipids and carotenoids, the primary pigments found in the hemolymph of female crabs [7]. However, no significant difference was observed in the L* values between the two groups (*p* > 0.05), suggesting that overall lightness did not differ substantially.

After steaming, the visual distinction between BC and NFMC becomes even more pronounced (Figure 1D). The body cavity of BC becomes filled with a bright yellow–orange buttery substance, and the red gonads typical of NFMC are absent. In contrast, NFMC retains fully developed red ovaries and white muscle tissue, with no buttery infiltration [1,6]. These visual differences underscore the distinctiveness of BC as a potential new product category. The visual characteristics of food play a significant role in influencing consumer perception, appetite, and purchasing decisions [18,19]. The unique appearance of BC, characterized by its distinct color—likely associated with carotenoid and lipid deposition—and the buttery texture it develops after cooking, makes it more appealing to consumers than NFMC [20,21]. These visual differences contribute to the growing popularity of BC in the market, as it stands out from the more conventional appearance of NFMC.

To quantitatively assess these visual differences after steaming, a structured sensory evaluation was conducted. As presented in Table S1, no statistically significant differences were observed between BC and NFMC in terms of external shell color (7.5 vs. 7.8), shell glossiness (8.2 vs. 8.3), or shell integrity (10.0 vs. 10.0) (*p* > 0.05), indicating a comparable overall shell appearance and physical condition between the two groups after steaming. However, BC outperformed NFMC in several muscle-related attributes, including color (8.0 vs. 6.6), oiliness (7.9 vs. 6.4), and firmness (8.3 vs. 7.7), indicating a visually more desirable internal muscle quality. Most notably, hepatopancreas-related attributes demonstrated the largest contrast between the two groups. BC exhibited significantly higher scores for color (8.5 vs. 6.1), oiliness (8.7 vs. 5.9), and firmness (8.0 vs. 6.3), all with *p* < 0.001. The visual impression of a plump, golden hepatopancreas and muscle likely conveys perceptions of richness and premium quality, aligning with previous findings that lipid-rich appearances in seafood products enhance perceived flavor and consumer willingness to pay [20,21]. In terms of overall visual appeal, BC was rated significantly higher than NFMC (8.2 ± 0.4 vs. 7.4 ± 0.5, *p* < 0.01). When asked to indicate their preferred sample based on appearance alone, 80% of panelists selected BC, while 20% favored NFMC. These findings indicate a modest but clear visual preference for BC, highlighting its potential market advantage based on appearance.

These results provide important insights for market segmentation and product positioning. Butter crab (BC), with its distinct glossy appearance and buttery hepatopancreas, is visually aligned with premium or novelty seafood products, suitable for high-end cuisine and export markets. In contrast, NFMC, with its familiar red gonads and clean white muscle, appeals more strongly to traditional and health-conscious consumers, especially in markets where conventional mud crab traits are valued.

### 3.2. Tissue Composition and Nutritional Value

As shown in Table 1, both NFMC and BC exhibited similar meat yield (MY) values, approximately 25%. However, significant differences were observed between the two crab types in terms of the gonadosomatic index (GSI) and hepatopancreas index (HSI) (*p* < 0.05). The GSI of NFMC was 12.92%, notably higher than the 1.35% observed in BC. In contrast, the HSI of BC (11.49%) was higher than that of NFMC (6.77%). These differences in tissue indices resulted in a higher total edible yield (TEY) in NFMC compared to BC. The distinct variations in GSI and HSI reflect the differing developmental strategies of these two crab types. The ovaries of BC exhibited hypogenetic gonadal tissues, occupying only a small portion of the body. This condition led to visible swelling of the hepatopancreas, which is normally covered by the ovaries. This alteration suggests that during maturation, energy and nutrients that would typically be allocated for gonadal development in BC are instead redirected toward the hepatopancreas, a key organ responsible for lipid and energy storage [1,3]. Such a shift in resource allocation could impact the overall flavor profile of BC [5].

In terms of nutritional composition, Table 1 reveals that BC muscle tissue contained significantly lower moisture levels compared to NFMC but higher lipid and protein content. No significant differences were found in ash content between the two crab types. The hepatopancreas of BC also showed higher lipid content and lower moisture content compared to NFMC (*p* < 0.05), likely due to the redirection of energy from gonadal development to lipid storage [3,22]. While no significant differences were observed in moisture or ash content between the ovaries of NFMC and BC, lipid and protein levels in the ovaries of NFMC were significantly higher. This finding supports the earlier observation that the ovaries of BC are less developed, leading to a lower accumulation of these essential nutrients [5,23,24]. The results of this study underscore how the distinct growth forms of NFMC and BC affect tissue indices, as well as the lipid and protein contents in the muscle, hepatopancreas, and ovaries. The lower GSI and underdeveloped gonads in BC suggest a reallocation of energy towards the hepatopancreas and muscle, contributing to the higher lipid content observed in these tissues compared to NFMC. This physiological adaptation likely enhances the growth and energy storage capacity of BC, while NFMC prioritizes gonadal development [23].

These differences in tissue composition may have important implications for marketability. The higher lipid content in the hepatopancreas and muscle of BC could make it more appealing for certain culinary applications, particularly those requiring rich, flavorful meat. In contrast, NFMC, with its higher GSI and well-developed gonads, may be more desirable for consumers seeking crab with more pronounced reproductive tissue. Understanding the differences in the nutritional value of both crabs is crucial for meeting consumer demand and ultimately affecting their market success.

### 3.3. Texture Analysis

**Figure 2:**
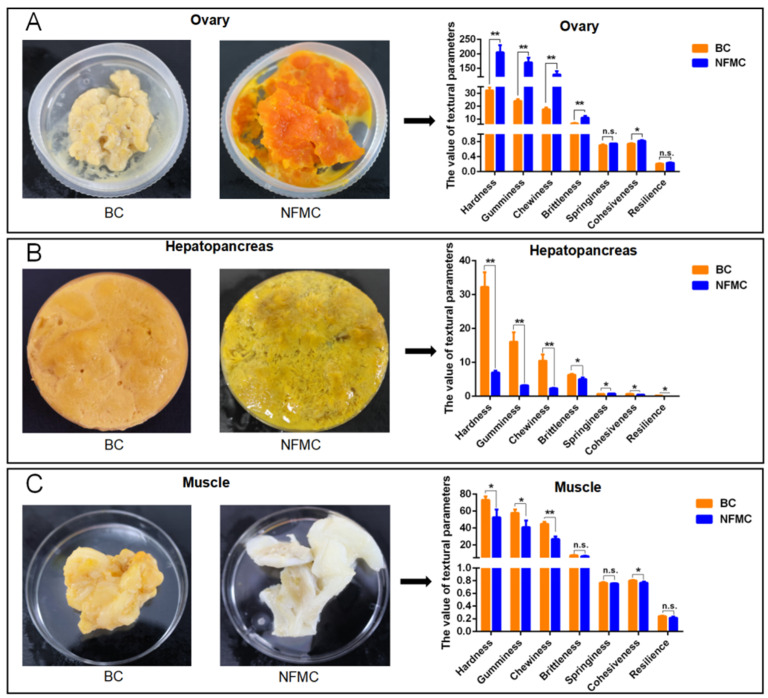
The texture of the muscle (**A**), hepatopancreas (**B**), and ovaries (**C**) of NFMC and BC. Data are expressed as mean ± standard deviation (*n* = 5). NFMC, normal female mud crab; BC, butter crab; n.s., no significant difference; * and ** mean *p* < 0.05 and *p* < 0.01, respectively.

The texture characteristics of muscle, hepatopancreas, and ovary tissues from NFMC and BC were analyzed using a texture analyzer, revealing notable differences between the two crab types (Figure 2). NFMC ovaries displayed significantly higher hardness, gumminess, chewiness, brittleness, and cohesiveness (*p* < 0.05) than BC ovaries, indicating that NFMC ovaries are firmer and more resilient. These differences are likely due to distinct nutritional compositions or structural properties [25,26]. In the ovaries, NFMC had higher lipid and protein levels, aligning with their firmer texture, while BC ovaries, being less developed, had lower lipid and protein content, resulting in a softer, more brittle texture. Furthermore, the hepatopancreas of NFMC was less elastic, exhibiting lower brittleness, springiness, and resilience compared to BC (*p* < 0.05). This suggests that NFMC hepatopancreas is more prone to deformation, possibly due to differences in lipid or protein content [27,28,29]. BC hepatopancreas contains higher lipid content and lower moisture compared to NFMC, which could explain its firmer texture. In addition, both muscle and hepatopancreas tissues from NFMC were softer, with lower hardness, gumminess, chewiness, and cohesiveness compared to BC (*p* < 0.05). Similarly, NFMC muscle has a higher moisture level, which may result in a softer texture. Conversely, BC muscle contains lower moisture levels but higher lipid and protein content compared to NFMC. This higher protein content likely contributes to the firmer texture of BC muscle.

These instrumental texture differences are also closely aligned with the sensory properties captured by electronic tongue and aroma profiling. For instance, the firmer texture of BC muscle correlates with enhanced umami and richness perceived in E-tongue analysis, as higher protein content and chewiness can amplify flavor release and prolong oral residence time, contributing to a more satisfying eating experience [30]. Similarly, the greater cohesiveness and resilience of BC hepatopancreas align with its creamier and less watery mouthfeel, which enhances sensory appeal [31]. In the ovaries, NFMC showed significantly higher hardness, gumminess, and chewiness (*p* < 0.05), which corresponds with its more developed gonads and higher nutrient accumulation. However, BC ovaries, while softer and more brittle due to lower structural protein and lipid content, may be perceived as more delicate or palatable by certain consumers. These contrasts highlight how textural variation—shaped by tissue-specific biochemical composition—directly contributes to the overall sensory experience.

Therefore, the integration of texture analysis with nutritional compositions and sensory profiling underscores the distinctive mouthfeel and consumer-relevant qualities of BC. This provides critical insight into how compositional differences translate into sensory attributes, reinforcing the superior perceived eating quality of BC and its potential for high-end market positioning.

### 3.4. Fatty Acid Analysis

**Figure 3:**
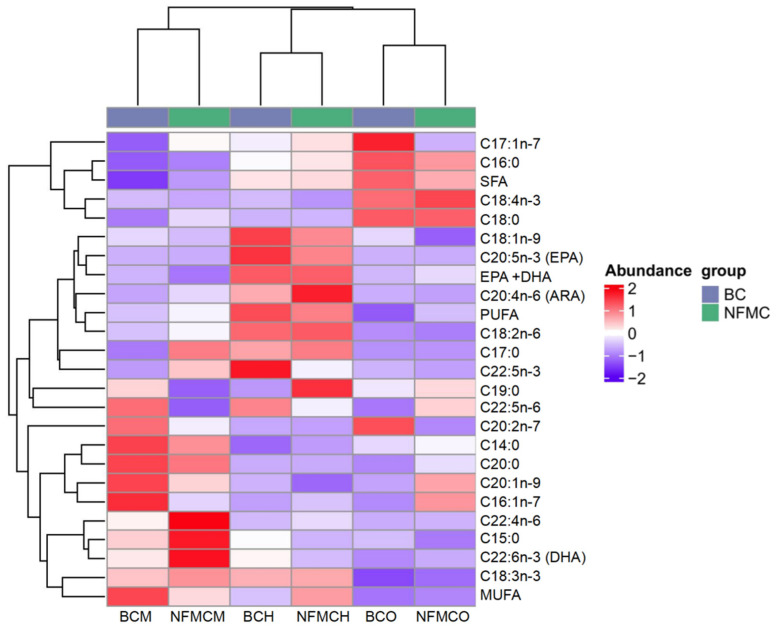
Heat map analysis of fatty acid contents (g/100 g total fatty acid) in the muscle, hepatopancreas, and ovaries of NFMC and BC. Data are expressed as mean ± standard deviation (*n* = 3). NFMC, normal female mud crab; BC, butter crab; M, muscle; H, hepatopancreas; O, ovary; SFA, saturated fatty acids; MUFA, monounsaturated fatty acids; PUFA, polyunsaturated fatty acids.

A total of 21 different fatty acids, including 7 saturated fatty acids (SFA), 4 monounsaturated fatty acids (MUFA), and 10 polyunsaturated fatty acids (PUFA), were identified in the muscle, hepatopancreas, and ovaries from NFMC and BC (Supplementary Table S2 and Figure 3). Among the SFAs, C16:0 and C18:0 were the predominant fatty acids in these tissues. Notably, the total SFA content in the muscle of BC was significantly lower than in NFMC, while the total SFA content in the ovaries of BC was significantly higher than in NFMC (*p* < 0.05). This suggests BC, due to its physiological characteristics and environmental factors, tends to accumulate more saturated fatty acids rather than converting them into the unsaturated fatty acids required for gonadal development [5,6,7,32].

Regarding MUFAs, the most abundant fatty acids were C16:1 and C18:1. The total MUFA content in the muscle of NFMC was significantly lower compared to BC, whereas the MUFA content in the hepatopancreas of NFMC was significantly higher than that of BC (*p* < 0.05). Interestingly, there were no significant differences in the MUFA content of the ovaries between the two species. This differential distribution of MUFAs might reflect variations in metabolic processes between the growth forms, with NFMC investing more MUFAs in the hepatopancreas for energy storage or cellular processes, while BC muscle shows a higher accumulation of MUFAs, likely contributing to its denser texture [33].

In terms of PUFAs, arachidonic acid (ARA), eicosapentaenoic acid (EPA), docosahexaenoic acid (DHA), and C22:5n-3 were the most abundant. Other PUFAs, such as C18:3, C18:4, and C22:4, were present in smaller quantities. For the two crab types, the total PUFA content in the ovaries of NFMC was significantly higher than in BC ovaries. In contrast, the total PUFA content in the hepatopancreas and muscle of NFMC was significantly lower than in BC (*p* < 0.05). This indicates that NFMC may prioritize the accumulation of PUFAs in the ovaries for oocyte development, while BC reallocates these fatty acids to the hepatopancreas and muscle, potentially for coping with environmental stress [3,5,22].

When examining the specific PUFAs, DHA and EPA levels were significantly higher in the ovaries of NFMC than in BC. Conversely, DHA and EPA levels in the muscle and hepatopancreas were significantly lower in NFMC than in BC, suggesting that BC accumulates more of these essential fatty acids in its muscle and hepatopancreas. The higher DHA and EPA content in muscle and hepatopancreas of BC indicates a better overall fatty acid nutritional profile. The total DHA + EPA content in the muscle and hepatopancreas was significantly higher in BC, potentially reflecting a reallocation of energy towards these tissues due to the underdeveloped ovaries in BC. This shift could support a pathological mechanism linked to environmental stress-induced vitellogenesis disruption in BC [8]. Specifically, impaired receptor-mediated endocytosis prevents hepatopancreas-synthesized vitellogenin (rich in DHA and EPA) from being transported into oocytes. This developmental abnormality in BC interrupts the directional mobilization of EPA and DHA toward gonadal tissues, leading to their pathological retention and accumulation in the hepatopancreas and muscle [5,8].

The observed differences in fatty acid distribution between NFMC and BC suggest that the nutritional quality of the fatty acids in the muscle and hepatopancreas of BC may be superior to that of NFMC. In contrast, NFMC seems to allocate more resources toward gonadal development, with the fatty acid composition in its ovaries potentially exhibiting higher nutritional quality than that of BC [7,23].

### 3.5. Free Amino Acids and Nucleotides Analysis

Flavor is a crucial factor influencing the marketability of mud crabs, and the levels of flavor compounds, primarily free amino acids and nucleotides, significantly impact the taste of these aquatic products [2,34]. A comparison between the flavor profiles of BC and NFMC reveals distinct differences in their free amino acid and nucleotide composition (Supplementary Tables S3 and S4), which directly contribute to their varying taste characteristics.

**Figure 4:**
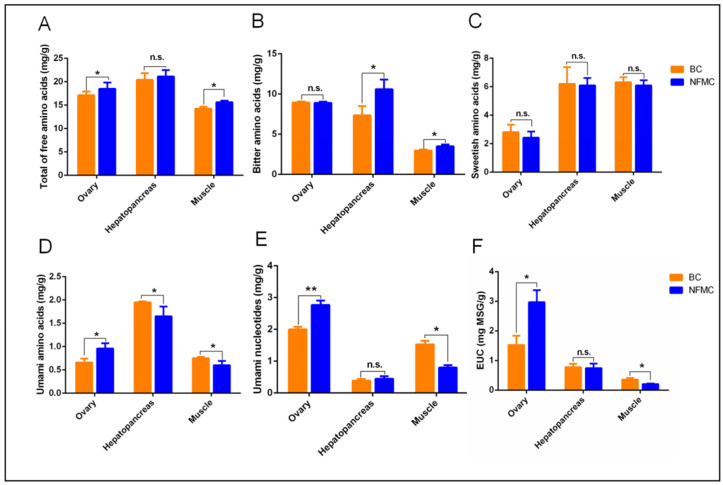
The results of free amino acids (**A**–**D**), umami nucleotides (**E**), and EUC (**F**) content in the muscle, hepatopancreas, and ovaries of NFMC and BC. Bitter amino acids include arginine, leucine, isoleucine, methionine, tyrosine, phenylalanine, valine, and tryptophan. Sweet amino acids include glycine, alanine, proline, threonine, and serine. Umami amino acids include aspartic acid and glutamic acid. Umami nucleotides include AMP, CMP, UMP, GMP, and IMP. Equivalent umami concentration (EUC) was devised for quantifying the MSG-like umami intensity [16]. Data are expressed as mean ± standard deviation (*n* = 3). NFMC, normal female mud crab; BC, butter crab; n.s., no significant difference; * and ** mean *p* < 0.05 and *p* < 0.01, respectively.

NFMC exhibited significantly higher levels of total free amino acids than BC in both muscle and ovaries, while no such differences were observed in the hepatopancreas (Figure 4A). Regarding bitter flavor, NFMC had higher concentrations of bitter amino acids in the muscle and hepatopancreas compared to BC. No significant differences were observed in the ovaries. (Figure 4B). For sweet-flavored amino acids, there were no significant differences between the two crabs in any of the tissues examined—muscle, hepatopancreas, or ovaries (Figure 4C). BC, on the other hand, had significantly higher levels of umami amino acids than NFMC in both muscle and hepatopancreas, but BC’s ovaries contained lower levels of umami amino acids compared to NFMC (Figure 4D). This suggests that BC’s muscle and hepatopancreas have a stronger umami profile with less bitterness, while NFMC’s ovaries provide a richer umami flavor. However, since the ovaries of BC are hypogenetic, the primary edible tissues of BC are the muscle and hepatopancreas, which further emphasizes the relevance of these tissues in terms of culinary applications.

The role of free nucleotides such as AMP, CMP, UMP, GMP, and IMP—along with the sodium salts of Asp and Glu—is critical in enhancing the umami taste. These compounds work synergistically to intensify the umami flavor [16,35]. In BC, the levels of umami nucleotides and equivalent umami intensity (EUC) in the muscle were significantly higher than those found in NFMC, suggesting that BC’s muscle offers a more robust umami profile. However, no significant differences in umami nucleotide and EUC content were found between the hepatopancreas of BC and NFMC, and NFMC’s ovaries contained significantly higher levels of these compounds than those of BC (Figure 4E,F). This indicates that NFMC’s ovaries contribute significantly to its umami flavor, which is several times more intense than that found in either the muscle or hepatopancreas.

These findings underscore the differing flavor profiles of NFMC and BC, which are influenced by their distinct metabolic priorities [5,6,36]. BC appears to allocate more energy toward the development of muscle and hepatopancreas, leading to higher concentrations of umami amino acids and nucleotides in these tissues. This makes BC’s muscle and hepatopancreas more appealing in terms of umami-rich flavor, ideal for culinary uses that emphasize savory taste. On the other hand, NFMC seems to prioritize reproductive tissue development, with its ovaries exhibiting higher umami intensity due to the accumulation of nucleotides and amino acids essential for reproduction.

### 3.6. Electronic Nose Analysis

**Figure 5:**
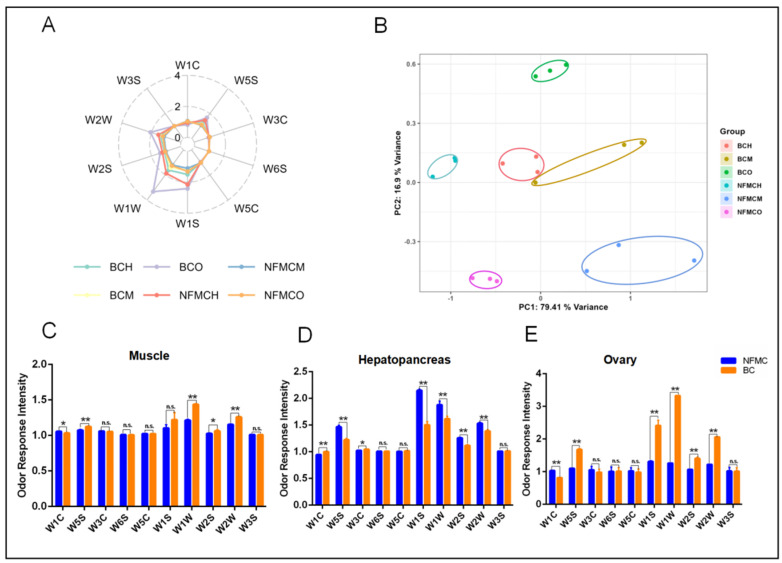
Radar map (**A**), principal component analysis (PCA) (**B**), and bar chart (**C**–**E**) of odor response intensity of the E-nose analysis in the muscle, hepatopancreas, and ovaries of NFMC and BC. Data are expressed as mean ± standard deviation (*n* = 3). NFMC, normal female mud crab; BC, butter crab; M, muscle; H, hepatopancreas; O, ovary; n.s., no significant difference; * and ** mean *p* < 0.05 and *p* < 0.01, respectively.

To investigate the aroma characteristics of NFMC and BC, E-nose analysis was used to compare the volatile compounds in their muscle, hepatopancreas, and ovary tissues. Figure 5A shows high responses from metal sensors W1W, W2W, W5S, W2S, and W1S, indicating the presence of volatile compounds like sulfides, nitrogen oxides, organic sulfides, and aromatic components in the edible tissues of both crab types [37,38].

Principal component analysis (PCA), depicted in Figure 5B, demonstrated that the first two principal components (PC1 and PC2) accounted for 96.31% of the variance, confirming the stability and reliability of the E-nose data. The PCA plot also showed clear separation between the tissue groups of NFMC and BC, suggesting distinct aroma profiles between the two crab types.

Comparing the sensor response values for each tissue, Figure 5C reveals that in the muscle, BC had significantly higher responses for W5S, W1W, W2S, and W2W than NFMC. This indicates that BC’s muscle has higher levels of sulfides, nitrogen oxides, and aromatic compounds, contributing to a more complex aroma. In the hepatopancreas (Figure 5D), BC showed lower responses for W5S, W1S, W1W, W2S, and W2W compared to NFMC but had a significantly higher response for W1C. This suggests that BC’s hepatopancreas contains fewer sulfides and aromatic compounds, resulting in a milder aroma than NFMC’s. For the ovaries (Figure 5E), BC again exhibited significantly higher responses for W5S, W1S, W1W, W2S, and W2W compared to NFMC, similar to the muscle tissue. This further supports the notion that BC’s ovaries have a stronger aroma, rich in sulfides, nitrogen oxides, and aromatic compounds. However, BC had lower W1C responses in both muscle and ovaries, indicating a lower level of aromatic compounds compared to NFMC.

The E-nose analysis reveals significant differences in the volatile compound profiles of the muscle, hepatopancreas, and ovaries of NFMC and BC, reflecting distinct aromatic characteristics between the two crab types. BC consistently exhibited higher levels of certain volatile compounds, such as sulfides, nitrogen oxides, and aromatic compounds, in both muscle and ovary tissues. This suggests that BC may have a more pronounced and complex aroma, potentially contributing to its unique flavor characteristics. These findings provide important insights into the aroma profiles of NFMC and BC and highlight the potential impact of tissue composition on their sensory characteristics. The distinct aroma profiles of the two crab types could influence consumer preference, with BC potentially offering a richer, more complex aroma, particularly in its muscle. However, these differences needed to be further verified using HS-SPME-GC-MS.

### 3.7. Electronic Tongue Analysis

**Figure 6:**
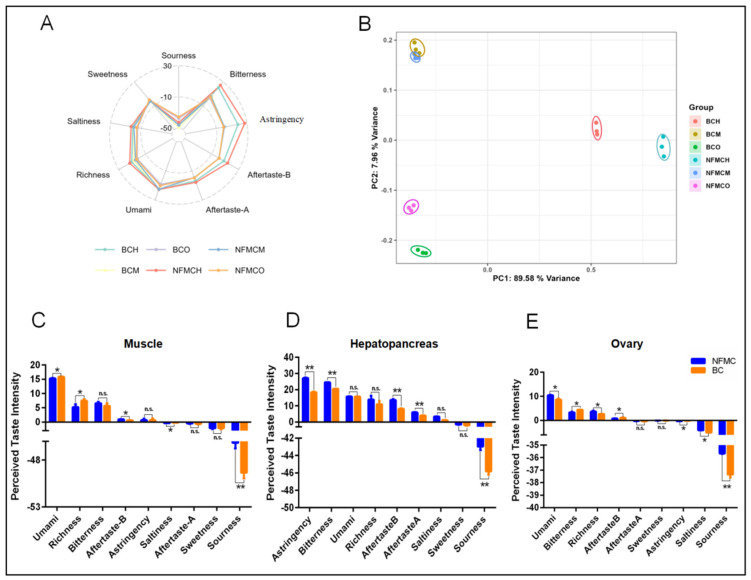
The results of radar map (**A**), principal component analysis (PCA) (**B**), and bar chart (**C**–**E**) of perceived taste intensity of the E-tongue analysis in the muscle, hepatopancreas, and ovaries of NFMC and BC. Data are expressed as mean ± standard deviation (*n* = 3). Abbreviations: aftertaste-A, aftertaste astringency; aftertaste-B, aftertaste bitterness; NFMC, normal female mud crab; BC, butter crab; M, muscle; H, hepatopancreas; O, ovary; n.s., no significant difference; * and ** mean *p* < 0.05 and *p* < 0.01, respectively.

The E-tongue is a device capable of converting electrical signals into taste signals, allowing for objective food taste profiling. It has a low sensory threshold and removes the subjective biases typically found in traditional sensory evaluation methods [39]. In this study, the E-tongue was used to analyze the taste characteristics of the muscle, hepatopancreas, and ovaries from NFMC and BC, with results visualized in Figure 6.

For the muscle, BC exhibited higher responses for umami, richness, and saltiness compared to NFMC but showed significantly lower responses for aftertaste-B and sourness. This finding aligns with the higher levels of umami amino acids, umami nucleotides, and EUC in BC’s muscle, suggesting a stronger umami flavor profile in BC. In contrast, the hepatopancreas showed a different pattern. BC had lower responses for astringency, bitterness, aftertaste-B, aftertaste-A, and sourness compared to NFMC. This was consistent with the higher concentrations of bitter amino acids found in NFMC’s hepatopancreas, indicating that NFMC’s hepatopancreas tends to have a more pronounced bitterness, while BC’s hepatopancreas is milder in bitterness and other taste characteristics. For the ovaries, BC showed lower responses for umami, richness, saltiness, astringency, and sourness compared to NFMC. However, BC’s ovaries had higher responses for bitterness and aftertaste-B. This suggests that while BC ovaries are richer in bitter compounds, they lack the umami and other savory notes found in NFMC ovaries. BC’s ovaries are underdeveloped (hypogenetic), confirming that the lower levels of umami amino acids, umami nucleotides, and EUC in BC ovaries contribute less to the overall flavor profile, especially in terms of umami. In contrast, NFMC’s more developed ovaries play a significant role in contributing to its umami flavor, highlighting the role of tissue development in the overall taste profile [5,40].

Principal component analysis (PCA) was performed to analyze the taste data further. The first two principal components (PC1: 89.58% and PC2: 7.96%) explained 97.54% of the total variance, demonstrating that these components capture most of the original data’s information. The PCA results revealed no significant overlap between NFMC and BC in the hepatopancreas and ovaries, but the muscle profiles of the two species showed partial overlap. This suggests that while the muscle tissues of both crab types share some similarities in taste, they still retain distinct differences, particularly in terms of umami and bitterness. In short, the analysis highlighted significant taste differences between the two crab types, especially in the muscle and ovaries, and emphasized the importance of tissue development in shaping taste.

### 3.8. Volatile Flavor Component Analysis

To assess the volatile flavor profiles of muscle, hepatopancreas, and ovary tissues in both NFMC and BC, volatile compounds were analyzed using HS-SPME-GC/MS. A total of fifty-seven major volatile compounds across the three edible tissues were identified, which were classified into nine categories: nine alkanes, fourteen aldehydes, six ketones, five alcohols, six aromatics, four esters, five sulfur-containing compounds, four nitrogen-containing compounds, and four others (Table 2). These volatile compounds are primarily formed through the degradation of lipids and proteins, with some serving as direct contributors to aroma, while others act as intermediates in flavor formation [38,41]. Notable differences were observed in both the composition and relative abundance of volatile compounds across tissues and between crab types. These findings were consistent with the earlier E-nose results, where BC samples demonstrated higher sensor responses, particularly in W5S, W1W, W2S, and W2W, which are sensitive to sulfur- and aromatic-containing volatiles.

Among the volatile compounds, aldehydes, alcohols, alkanes, and esters were the most prevalent, particularly in muscle tissues. Alkanes and esters, though abundant, are generally considered to have minimal olfactory contribution due to their high odor thresholds [6,34,42]. In contrast, aldehydes possess relatively low odor thresholds and are widely recognized as key contributors to the aroma of aquatic products [34,43]. This observation aligns with previous studies indicating that aldehydes, particularly those derived from polyunsaturated fatty acid (PUFA) oxidation, such as hexanal, nonanal, and decanal, play dominant roles in crustacean flavor development [34,42,44].

In muscle tissue, the total aldehyde content was significantly higher in BC (35.06 g/100 g total volatiles) than in NFMC (24.50 g/100 g). BC muscle contained elevated levels of pentanal, hexanal, octanal, nonanal, and decanal—compounds commonly associated with pleasant green, fatty, and fruity aromas originating from lipid oxidation [6]. This finding is in accordance with earlier data showing higher lipid and PUFA levels in BC muscle, reinforcing the link between lipid composition and aldehyde formation. Conversely, the muscle of NFMC exhibited higher levels of benzaldehyde, which is known to impart pungent or bitter off-notes [6,43], potentially diminishing its sensory appeal. Furthermore, benzeneacetaldehyde—characterized by floral and fruity nuances—was exclusively detected in BC muscle, likely contributing positively to its overall aroma profile [45]. Similar trends were observed in the hepatopancreas. The total aldehyde content was higher in BC (34.37 g/100 g) than in NFMC (28.98 g/100 g), with BC samples showing greater concentrations of pentanal, hexanal, and nonanal. These aldehydes, often associated with freshness and richness, likely enhance the sensory appeal of BC hepatopancreas [34,42]. The elevated aldehyde levels in BC are consistent with its higher lipid content, further supporting the lipid-derived origin of these volatile compounds [46]. Interestingly, an inverse pattern was observed in the ovary tissue. NFMC ovaries exhibited a significantly higher total aldehyde content (53.00 g/100 g) compared to BC (38.90 g/100 g), with greater concentrations of key aldehydes including nonanal, hexanal, benzeneacetaldehyde, (E,Z)-2,6-nonadienal, and hexadecanal. These compounds are known for their floral, fruity, and fatty notes, and their abundance in NFMC ovaries likely contributes to a richer and more complex sensory experience. These findings corroborate earlier observations that NFMC ovaries are more developed and nutrient-rich, supporting their enhanced flavor potential.

From a market perspective, the higher aldehyde concentrations and richer volatile profiles in the muscle and hepatopancreas of BC may contribute to its more favorable aroma characteristics, aligning with consumer preferences for intensely flavored seafood products. Previous consumer studies have shown that flavor intensity and pleasant aroma are key determinants of purchase intent in crustaceans and other aquatic products [47]. Therefore, the superior volatile profile of BC tissues may partly explain its premium market position and higher commercial value. Conversely, the greater flavor complexity observed in NFMC ovaries suggests potential for targeted marketing, particularly in regions such as East Asia, where gonadal tissues are highly prized for their sensory qualities during seasonal consumption peaks [37,48]. These differences highlight how developmental strategy not only shapes nutritional profiles but also determines sensory and market-related traits.

## 4. Conclusions

This study conducted a comprehensive comparison of the nutritional composition, texture properties, and sensory profiles of BC and NFMC, revealing distinct differences between the two phenotypes. BC exhibited higher lipid content, firmer tissue texture, and a richer profile of umami-related amino acids in both muscle and hepatopancreas. Additionally, BC muscle demonstrated a more complex and diverse aroma profile compared to NFMC. These integrated findings underscore the superior nutritional and sensory attributes of BC, supporting its potential as a high-value seafood product. The results offer valuable implications for the refinement of aquaculture practices, development of consumer-oriented marketing strategies, and promotion of product differentiation within the *S. paramamosain* industry.

## Figures and Tables

**Table 1:** Tissue indices, total edible yields, and proximate compositions of NFMC and BC.

	NFMC	BC
Tissue indices and total edible yields (%)		
MY	26.04 ± 1.57 ^a^	25.04 ± 0.82 ^a^
HSI	6.77 ± 0.45 ^a^	11.49 ± 1.63 ^b^
GSI	12.92 ± 1.36 ^b^	1.35 ± 0.31 ^a^
TEY	45.73 ± 1.78 ^b^	37.88 ± 1.96 ^a^
Proximate compositions in the muscle (g/100 g wet weight)		
Moisture	76.12 ± 0.92 ^b^	73.32 ± 0.86 ^a^
Protein	19.73 ± 0.25 ^a^	21.65 ± 0.14 ^b^
Lipid	0.57 ± 0.08 ^a^	0.77 ± 0.06 ^b^
Ash	1.84 ± 0.04 ^a^	1.77 ± 0.05 ^a^
Proximate compositions in the hepatopancreas (g/100 g wet weight)		
Moisture	58.52 ± 0.76 ^b^	54.33 ± 1.17 ^a^
Protein	14.81 ± 0.91 ^a^	13.55 ± 1.09 ^a^
Lipid	17.14 ± 0.72 ^a^	22.14 ± 1.68 ^b^
Ash	2.01 ± 0.22 ^a^	1.97 ± 0.52 ^a^
Proximate compositions in the ovaries (g/100 g wet weight)		
Moisture	50.45 ± 0.32 ^a^	52.18 ± 0.89 ^a^
Protein	32.62 ± 0.75 ^b^	28.50 ± 1.12 ^a^
Lipid	2.51 ± 0.39 ^b^	1.88 ± 0.26 ^a^
Ash	1.98 ± 0.45 ^a^	1.86 ± 0.37 ^a^

Note: HSI(%) = hepatopancreas weight/body weight × 100; MY(%) = meat weight/body weight × 100; GSI(%) = gonads wet weight/body wet weight × 100; TEY(%) = MY + HIS + GSI. Data are expressed as mean ± standard deviation (*n* = 4). NFMC: normal female mud crab; BC: butter crab; HSI: hepatopancreas index; MY: meat yield; GSI: gonadosomatic index; TEY: total edible yield. Data in the same row with different superscripts are significantly different (*p* < 0.05).

**Table 2:** Major volatile compound contents in the ovary, hepatopancreas, and muscle of NFMC and BC (g/100 g total volatile compounds).

Compounds	Muscle		Hepatopancreas		Ovary	
	NFMC	BC	NFMC	BC	NFMC	BC
Alkanes (9)						
1,3,5,7-Cyclooctatetraene	1.03 ± 0.05 ^a^	3.18 ± 0.11 ^b^	4.87 ± 0.26 ^b^	2.96 ± 0.17 ^a^	1.21 ± 0.07 ^b^	- ^a^
α-Phellandrene	1.43 ± 0.10 ^a^	2.95 ± 0.13 ^b^	1.89 ± 0.11 ^a^	3.01 ± 0.23 ^b^	4.37 ± 0.26 ^b^	3.11 ± 0.19 ^a^
Limonene	3.11 ± 0.17 ^a^	5.01 ± 0.28 ^b^	4.44 ± 0.31 ^a^	6.86 ± 0.42 ^b^	2.64 ± 0.10 ^b^	1.81 ± 0.09 ^a^
Dodecane	1.02 ± 0.07 ^a^	1.45 ± 0.09 ^b^	0.23 ± 0.05 ^a^	0.18 ± 0.03 ^a^	0.21 ± 0.01 ^b^	- ^a^
Tridecane	0.29 ± 0.02 ^a^	0.37 ± 0.11 ^a^	0.20 ± 0.01 ^a^	0.48 ± 0.02 ^b^	0.31 ± 0.03 ^a^	1.16 ± 0.10 ^b^
Tetradecane	0.99 ± 0.12 ^a^	0.75 ± 0.14 ^a^	1.18 ± 0.05 ^b^	0.63 ± 0.03 ^a^	3.04 ± 0.15 ^a^	4.12 ± 0.23 ^b^
Pentadecane	1.51 ± 0.12 ^b^	0.35 ± 0.02 ^a^	0.36 ± 0.09 ^a^	0.47 ± 0.12 ^a^	0.82 ± 0.04 ^a^	1.13 ± 0.10 ^b^
Hexadecane	1.13 ± 0.07 ^b^	0.42 ± 0.02 ^a^	- ^a^	- ^a^	0.93 ± 0.06 ^b^	- ^a^
2,6,10,14-Tetramethylpentadecane	2.47 ± 0.09 ^b^	1.35 ± 0.07 ^a^	0.26 ± 0.03 ^a^	0.18 ± 0.05 ^a^	2.21 ± 0.09 ^a^	3.24 ± 0.11 ^b^
Subtotal	12.98 ± 0.31 ^a^	15.83 ± 0.57 ^b^	13.43 ± 0.71 ^a^	14.77 ± 0.43 ^a^	15.74 ± 0.32 ^b^	14.57 ± 0.26 ^a^
Aldehydes (14)						
3-Methylbutanal	2.51 ± 0.10 ^b^	1.73 ± 0.06 ^a^	2.19 ± 0.11 ^b^	1.63 ± 0.06 ^a^	2.11 ± 0.09 ^b^	0.92 ± 0.05 ^a^
Pentanal	4.14 ± 0.18 ^a^	7.12 ± 0.27 ^b^	10.07 ± 0.43 ^a^	12.42 ± 0.54 ^b^	19.9 ± 0.93 ^b^	15.16 ± 0.79 ^a^
Hexanal	1.17 ± 0.11 ^a^	3.73 ± 0.21 ^b^	2.92 ± 0.18 ^a^	4.82 ± 0.25 ^b^	9.16 ± 0.40 ^b^	6.47 ± 0.37 ^a^
Benzaldehyde	6.67 ± 0.26 ^b^	3.54 ± 0.17 ^a^	2.31 ± 0.13 ^b^	1.65 ± 0.07 ^a^	7.24 ± 0.43 ^a^	10.36 ± 0.78 ^b^
Octanal	0.49 ± 0.04 ^a^	1.25 ± 0.06 ^b^	1.03 ± 0.09 ^a^	1.85 ± 0.04 ^b^	1.35 ± 0.11 ^b^	0.45 ± 0.08 ^a^
Benzeneacetaldehyde	- ^a^	0.21 ± 0.02 ^b^	0.53 ± 0.03 ^a^	1.52 ± 0.11 ^b^	3.16 ± 0.13 ^b^	1.12 ± 0.08 ^a^
(E)-2-Octenal	-	-	0.69 ± 0.04 ^b^	0.16 ± 0.01 ^a^	3.65 ± 0.12 ^b^	1.88 ± 0.09 ^a^
Nonanal	5.72 ± 0.25 ^a^	7.34 ± 0.21 ^b^	5.67 ± 0.36 ^a^	7.91 ± 0.53 ^b^	2.31 ± 0.16 ^b^	1.08 ± 0.04 ^a^
(E, Z)-2,6-Nonadienal	- ^a^	- ^a^	0.20 ± 0.02 ^b^	- ^a^	0.47 ± 0.04 ^b^	0.18 ± 0.01 ^a^
(E)-2-Nonenal	- ^a^	- ^a^	0.38 ± 0.02 ^b^	0.18 ± 0.01 ^a^	0.43 ± 0.02 ^b^	0.16 ± 0.01 ^a^
Decanal	1.43 ± 0.12 ^a^	3.51 ± 0.23 ^b^	0.63 ± 0.03 ^b^	0.21 ± 0.01 ^a^	0.52 ± 0.01 ^b^	0.23 ± 0.01 ^a^
(E, E)-2,4-Decadienal	0.43 ± 0.05 ^a^	0.34 ± 0.11 ^a^	- ^a^	0.60 ± 0.02 ^b^	1.15 ± 0.05 ^b^	0.52 ± 0.03 ^a^
Pentadecanal	1.42 ± 0.10 ^a^	4.87 ± 0.23 ^b^	0.92 ± 0.05 ^b^	0.16 ± 0.01 ^a^	0.31 ± 0.02 ^b^	- ^a^
Hexadecanal	0.52 ± 0.03 ^a^	1.42 ± 0.04 ^b^	1.44 ± 0.10 ^a^	1.26 ± 0.18 ^a^	1.24 ± 0.10 ^b^	0.37 ± 0.02 ^a^
Subtotal	24.50 ± 0.46 ^a^	35.06 ± 0.52 ^b^	28.98 ± 0.45 ^a^	34.37 ± 0.58 ^b^	53.00 ± 0.68 ^b^	38.90 ± 0.95 ^a^
Ketones (6)						
Acetone	0.31 ± 0.07 ^a^	0.24 ± 0.03 ^a^	- ^a^	0.33 ± 0.01 ^b^	0.52 ± 0.02 ^b^	- ^a^
2-Heptanone	2.11 ± 0.07 ^b^	1.76 ± 0.06 ^a^	0.26 ± 0.02 ^a^	0.54 ± 0.04 ^b^	0.51 ± 0.03 ^a^	1.57 ± 0.09 ^b^
2-Nonanone	- ^a^	0.21 ± 0.01 ^b^	0.16 ± 0.01 ^a^	0.30 ± 0.02 ^b^	- ^a^	0.18 ± 0.01 ^b^
1-Decen-3-one	- ^a^	- ^a^	1.02 ± 0.12 ^a^	1.32 ± 0.14 ^a^	0.16 ± 0.01 ^a^	2.69 ± 0.16 ^b^
2-Methyl-3-heptanone,	0.21 ± 0.01 ^a^	0.85 ± 0.06 ^b^	0.74 ± 0.04 ^a^	1.65 ± 0.11 ^b^	0.52 ± 0.03 ^b^	0.32 ± 0.02 ^a^
3-Decanone	4.86 ± 0.25 ^a^	6.48 ± 0.36 ^b^	5.56 ± 0.27 ^b^	1.98 ± 0.09 ^a^	0.85 ± 0.05 ^a^	1.73 ± 0.11 ^b^
Subtotal	7.49 ± 0.12 ^a^	9.54 ± 0.11 ^b^	7.74 ± 0.15 ^b^	6.12 ± 0.13 ^a^	2.56 ± 0.05 ^a^	6.49 ± 0.12 ^b^
Alcohols (5)						
1-Penten-3-ol	0.82 ± 0.15 ^a^	0.64 ± 0.13 ^a^	0.85 ± 0.16 ^a^	0.72 ± 0.05 ^a^	1.15 ± 0.04 ^a^	2.94 ± 0.10 ^b^
1-Octen-3-ol	0.37 ± 0.02 ^a^	0.59 ± 0.03 ^b^	1.20 ± 0.10 ^b^	0.51 ± 0.04 ^a^	1.66 ± 0.11 ^a^	2.45 ± 0.12 ^b^
1-Octanol	0.22 ± 0.01 ^a^	1.13 ± 0.08 ^b^	1.06 ± 0.05 ^a^	2.03 ± 0.13 ^b^	1.71 ± 0.07 ^a^	1.87 ± 0.18 ^a^
3-Decanol	3.41 ± 0.17 ^a^	5.14 ± 0.26 ^b^	3.40 ± 0.16 ^a^	4.91 ± 0.21 ^b^	4.15 ± 0.24 ^a^	5.01 ± 0.36 ^b^
Benzyl Alcohol	1.47 ± 0.11 ^b^	0.25 ± 0.01 ^a^	0.18 ± 0.01 ^a^	1.04 ± 0.10 ^b^	0.35 ± 0.03 ^a^	0.75 ± 0.05 ^b^
Subtotal	6.29 ± 0.13 ^a^	7.75 ± 0.10 ^b^	6.69 ± 0.12 ^a^	9.21 ± 0.14 ^b^	9.02 ± 0.18 ^a^	13.02 ± 0.26 ^b^
Aromatics (6)						
Toluene	0.66 ± 0.15 ^a^	0.47 ± 0.12 ^a^	12.40 ± 1.12 ^b^	5.64 ± 0.36 ^a^	- ^a^	0.17 ± 0.01 ^b^
Ethylbenzene	0.14 ± 0.01 ^a^	0.71 ± 0.03 ^b^	0.59 ± 0.02 ^a^	0.72 ± 0.03 ^b^	- ^a^	- ^a^
1, 3-dimethyl-Benzene	2.12 ± 0.09 ^a^	3.70 ± 0.015 ^b^	5.40 ± 0.43 ^b^	2.67 ± 0.16 ^a^	2.56 ± 0.11 ^a^	7.43 ± 0.40 ^b^
*p*-Xylene	2.16 ± 0.10 ^b^	0.45 ± 0.03 ^a^	2.09 ± 0.25 ^a^	2.85 ± 0.37 ^a^	- ^a^	- ^a^
Styrene	2.33 ± 0.11 ^a^	3.45 ± 0.18 ^b^	1.55 ± 0.07 ^a^	3.10 ± 0.11 ^b^	- ^a^	- ^a^
1-Methyl-2-(1-methylethyl)-Benzene	0.63 ± 0.05 ^b^	- ^a^	2.14 ± 0.20 ^a^	4.62 ± 0.25 ^b^	0.65 ± 0.03 ^b^	0.31 ± 0.02 ^a^
Subtotal	8.04 ± 0.15 ^a^	8.78 ± 0.18 ^b^	24.17 ± 0.42 ^b^	19.6 ± 0.55 ^a^	3.21 ± 0.07 ^a^	7.91 ± 0.21 ^b^
Esters (4)						
Dibutyl phthalate	0.71 ± 0.04 ^b^	0.24 ± 0.02 ^a^	- ^a^	- ^a^	- ^a^	0.16 ± 0.01 ^b^
Hexadecanoic acid, ethyl ester	15.24 ± 1.31 ^b^	2.31 ± 0.20 ^a^	1.23 ± 0.11 ^a^	1.82 ± 0.10 ^b^	2.15 ± 0.12 ^b^	1.04 ± 0.04 ^a^
Ethyl Oleate	5.31 ± 0.26 ^b^	1.15 ± 0.13 ^a^	1.11 ± 0.10 ^b^	0.71 ± 0.05 ^a^	2.12 ± 0.14 ^b^	0.81 ± 0.06 ^a^
Linoleic acid ethyl ester	0.86 ± 0.05 ^b^	0.45 ± 0.03 ^a^	- ^a^	- ^a^	- ^a^	- ^a^
Subtotal	22.12 ± 0.35 ^b^	4.15 ± 0.16 ^a^	2.34 ± 0.10 ^a^	2.53 ± 0.0.7 ^b^	4.27 ± 0.08 ^b^	2.01 ± 0.05 ^a^
S-containing (5)						
Dimethyl sulfide	0.22 ± 0.04 ^a^	0.34 ± 0.12 ^a^	0.65 ± 0.03 ^b^	0.32 ± 0.02 ^a^	- ^a^	1.13 ± 0.11 ^b^
Dimethyl trisulfide	0.26 ± 0.03 ^a^	0.32 ± 0.06 ^a^	0.52 ± 0.03 ^a^	0.46 ± 0.08 ^a^	- ^a^	2.32 ± 0.17 ^b^
Vinyl ethyl sulfoxide	- ^a^	- ^a^	0.47 ± 0.02 ^b^	- ^a^	- ^a^	0.36 ± 0.03 ^b^
Ethylvinyl sulfde	0.42 ± 0.02 ^b^	0.17 ± 0.01 ^a^	0.23 ± 0.02 ^b^	- ^a^	0.18 ± 0.01 ^a^	0.56 ± 0.04 ^b^
Carbamodithioic acid, diethyl-, methyl ester	- ^a^	1.12 ± 0.09 ^b^	- ^a^	- ^a^	- ^a^	0.81 ± 0.05 ^b^
Subtotal	0.90 ± 0.05 ^a^	1.95 ± 0.10 ^b^	1.87 ± 0.05 ^b^	0.78 ± 0.04 ^a^	0.18 ± 0.01 ^a^	5.18 ± 0.21 ^b^
N-containing (4)						
Trimethylamine	2.14 ± 0.17 ^b^	1.87 ± 0.10 ^a^	0.52 ± 0.05 ^b^	0.21 ± 0.02 ^a^	- ^a^	0.28 ± 0.03 ^b^
Pyridine	1.42 ± 0.18 ^a^	1.35 ± 0.11 ^a^	1.53 ± 0.16 ^a^	1.21 ± 0.24 ^a^	0.52 ± 0.03 ^a^	1.36 ± 0.10 ^b^
3-Methyl-Cinnoline	0.46 ± 0.03 ^b^	- ^a^	- ^a^	- ^a^	- ^a^	0.05 ± 0.01 ^b^
4-Octadecyl-morpholine	3.10 ± 0.13 ^b^	0.11 ± 0.01 ^a^	- ^a^	- ^a^	- ^a^	1.03 ± 0.04 ^b^
Subtotal	7.12 ± 0.21 ^b^	3.33 ± 0.11 ^a^	2.05 ± 0.10 ^b^	1.42 ± 0.13 ^a^	0.52 ± 0.03 ^a^	2.72 ± 0.08 ^b^
Others (4)						
1-Methoxy-4-(1-propenyl)-Benzene	0.80 ± 0.07 ^a^	1.91 ± 0.10 ^b^	- ^a^	- ^a^	- ^a^	- ^a^
Butylated Hydroxytoluene	0.32 ± 0.12 ^a^	0.26 ± 0.03 ^a^	- ^a^	- ^a^	- ^a^	- ^a^
Decyl-oxirane	- ^a^	- ^a^	0.35 ± 0.02 ^b^	0.22 ± 0.01 ^a^	0.24 ± 0.02 ^a^	0.85 ± 0.06 ^b^
Hexadecyl-oxirane	0.08 ± 0.01 ^a^	1.97 ± 0.13 ^b^	0.32 ± 0.01 ^a^	0.84 ± 0.06 ^b^	0.05 ± 0.01 ^a^	0.17 ± 0.01 ^b^
Subtotal	1.20 ± 0.05 ^a^	4.14 ± 0.13 ^b^	0.67 ± 0.04 ^a^	1.06 ± 0.06 ^b^	0.29 ± 0.02 ^a^	1.02 ± 0.05 ^b^

Note: NFMC, normal female mud crab; BC, butter crab; -, not detected. Data are presented as mean values ± standard deviation (SD) obtained from three independent analyses (*n* =3). Different superscript letters in a row of the particular tissue indicate a significant difference (*p* < 0.05).

## Data Availability

The original contributions presented in this study are included in the article/Supplementary Materials. Further inquiries can be directed to the corresponding author.

## Supplementary Materials

The following supporting information can be downloaded at: https://www.mdpi.com/article/10.3390/foods14122101/s1. Table S1: Visual sensory evaluation scores (mean ± SD) and panelist preference (%) for NFMC and BC after cooking; Table S2: Fatty acid contents in the muscle, hepatopancreas, and ovaries of NFMC and BC (g/100 g total fatty acid); Table S3: The content of FAAs in the muscle, hepatopancreas and ovaries of NFMC and BC (mg/g wet weight); Table S4: The content of free nucleotides in the muscle, hepatopancreas, and ovaries of NFMC and BC (mg/100 g).
