# Supplementary material for: Comparative Analysis of Nutritional, Textural, and Sensory Attributes of Butter Crab and Normal Female Mud Crab (Scylla paramamosain): Insights for Market Positioning and Consumer Preference
Source: Foods. 2025 Jun 15;14(12):2101. doi: 10.3390/foods14122101 (PMC12192415; doi:10.3390/foods14122101)
Supplement: Supplementary file 1 [file foods-14-02101-s001.zip › foods-3663737-supplementary.pdf]

Supplementary information

Table S1

Visual sensory evaluation scores (mean ± SD) and panelist preference (%) for NFMC and BC after cooking

**Visual Attribute**	**NFMC**	**BC**	**Significance (*p*-value)**
External shell color	7.5 ± 0.5	7.8 ± 0.6	n.s. ($p > 0.05$)
Shell glossiness	8.2 ± 0.6	8.3 ± 0.8	n.s. ($p > 0.05$)
Shell integrity	10.0 ± 0.0	10.0 ± 0.0	n.s. ($p > 0.05$)
Muscle color	6.6 ± 0.8	8.0 ± 0.4	$p < 0.01$
Muscle oiliness	6.4 ± 0.9	7.9 ± 0.5	$p < 0.01$
Muscle firmness	7.7 ± 0.6	8.3 ± 0.4	$p < 0.05$
Hepatopancreas color	6.1 ± 0.7	8.5 ± 0.5	$p < 0.001$
Hepatopancreas oiliness	5.9 ± 0.8	8.7 ± 0.3	$p < 0.001$
Hepatopancreas firmness	6.3 ± 0.6	8.0 ± 0.4	$p < 0.001$
Overall visual appeal	7.4 ± 0.5	8.2 ± 0.4	$p < 0.01$
Preferred sample (% of panelists)	20%	80%	—

Note: Data represent mean ± standard deviation from 10 trained panelists. n.s.: not significant.

Table S2

Fatty acids contents in the muscle, hepatopancreas and ovaries of NFMC and BC (g/100 g total fatty acid)

Fatty acid	Muscle		Hepatopancreas		Ovaries	
	NFMC	BC	NFMC	BC	NFMC	BC
C14:0	2.31 ± 0.31[a]	2.56 ± 0.92[a]	3.91 ± 0.15[a]	4.56 ± 0.32[b]	1.51 ± 0.34[a]	2.56 ± 0.52[b]
C15:0	1.21 ± 0.13[b]	0.82 ± 0.07[a]	2.51 ± 0.23[a]	2.42 ± 0.24[a]	0.32 ± 0.07[a]	0.43 ± 0.15[b]
C16:0	16.05 ± 0.95[b]	14.13 ± 0.31[a]	23.70 ± 1.55[a]	22.24 ± 1.21[a]	27.72 ± 1.69[a]	31.15 ± 2.52[b]
C17:0	3.24 ± 0.18[b]	2.52 ± 0.16[a]	2.04 ± 0.06[a]	2.32 ± 0.15[b]	1.81 ± 0.13[a]	2.08 ± 0.53[a]
C18:0	8.17 ± 0.38[b]	5.98 ± 0.32[a]	7.35 ± 0.38[a]	7.27 ± 0.22[a]	12.76 ± 2.07[a]	12.88 ± 1.68[a]
C19:0	1.34 ± 0.11[b]	0.05 ± 0.01 [a]	1.34 ± 0.01[a]	1.15 ± 0.11 [a]	0.18 ± 0.01[a]	0.17 ± 0.05[a]
C20:0	0.14 ± 0.02[a]	0.15 ± 0.01[a]	0.65 ± 0.02[a]	0.85 ± 0.03[b]	0.14 ± 0.02[a]	0.15 ± 0.02[a]
∑SFA	32.46 ± 3.68[b]	26.21 ± 2.42[a]	41.50 ± 1.04[a]	40.81 ± 2.42[a]	44.44 ± 2.13[a]	49.42 ± 2.24[b]
C16:1n-7	8.18 ± 0.28[a]	7.58 ± 0.35[a]	11.38 ± 0.38[b]	9.68 ± 0.24[a]	7.51 ± 1.13[a]	7.68 ± 0.26[a]
C17:1n-7	1.03 ± 0.12[b]	0.33 ± 0.15[a]	1.13 ± 0.04[b]	0.93 ± 0.05[a]	0.68 ± 0.03[a]	1.93 ± 0.37[b]
C18:1n-9	23.84 ± 1.27[a]	27.34 ± 2.67[b]	14.48 ± 0.73[b]	12.15 ± 0.47[a]	18.49 ± 0.64[a]	17.16 ± 1.23[a]
C20:1n-9	1.07 ± 0.24[a]	2.67 ± 0.42[b]	8.71 ± 0.62[a]	8.82 ± 0.26[a]	3.64 ± 0.23[b]	2.75 ± 0.36[a]
∑MUFA	34.12 ± 0.86[a]	37.92 ± 1.76[b]	35.70 ± 0.47[b]	31.58 ± 0.21[a]	30.02 ± 0.25[a]	29.52 ± 1.89[a]
C18:2n-6	5.53 ± 0.48[b]	2.71 ± 0.08[a]	2.13 ± 0.14[b]	1.74 ± 0.12[a]	1.67 ± 0.29[a]	1.58 ± 0.22[a]
C18:3n-3	0.13 ± 0.01[a]	0.56 ± 0.11[b]	0.91 ± 0.11[b]	0.25 ± 0.01[a]	0.55 ± 0.07[a]	0.42 ± 0.14[a]
C18:4n-3	0.05 ± 0.01[a]	0.06 ± 0.07[a]	0.04 ± 0.01[a]	0.06 ± 0.02[a]	0.19 ± 0.16[a]	0.17 ± 0.07[a]
C20:2n-7	1.42 ± 0.33[a]	1.02 ± 0.32 [a]	1.24 ± 0.33[a]	1.87 ± 0.42 [a]	1.03 ± 0.16[a]	1.08 ± 0.34[a]
C20:4n-6 (ARA)	5.34 ± 0.31[a]	7.24 ± 0.26[b]	4.44 ± 0.35[a]	4.53 ± 0.17[a]	4.16 ± 0.31[a]	7.60 ± 1.40[b]
C20:5n-3 (EPA)	8.79 ± 0.32[a]	11.59 ± 0.81[b]	4.98 ± 0.21[a]	6.46 ± 0.15[b]	9.73 ± 0.54[a]	6.15 ± 0.32[a]

Table S2 (continued)

**Fatty acid**	**Muscle**		**Hepatopancreas**		**Ovaries**	
	**NFMC**	**BC**	**NFMC**	**BC**	**NFMC**	**BC**
C22:4n-6	0.88 ± 0.04[a]	0.78 ± 0.17[a]	1.17 ± 0.11[a]	1.28 ± 0.14[a]	0.77 ± 0.17[a]	0.56 ± 0.24[a]
C22:5n-3	7.20 ± 1.47[a]	3.28 ± 0.37[a]	1.64 ± 0.22[a]	3.05 ± 0.69[b]	1.35 ± 0.32[b]	0.74 ± 0.21[a]
C22:5n-6	2.16 ± 0.13[b]	1.86 ± 0.11[a]	2.02 ± 0.18[a]	1.97 ± 0.17[a]	0.62 ± 0.06[b]	0.42 ± 0.16[a]
C22:6n-3 (DHA)	1.92 ± 0.39[a]	6.77 ± 1.12[b]	4.23 ± 0.12[a]	6.40 ± 0.24[b]	5.17 ± 0.65[b]	2.34 ± 1.03[a]
∑PUFA	33.42 ± 1.09[a]	35.87 ± 0.69[b]	22.80 ± 0.58[a]	27.61 ± 0.32[b]	25.24 ± 0.55[b]	21.06 ± 1.12[a]
EPA + DHA	10.71 ± 0.49[a]	18.36 ± 1.69[b]	9.21 ± 0.33[a]	12.86 ± 0.23[b]	15.20 ± 0.57[b]	8.49 ±0.83[a]

Note: Data are mean ± standard deviation (n = 4). NFMC, normal female mud crab; BC, butter crab; SFA, saturated fatty acid; MUFA, monounsaturated fatty acid; PUFA, polyunsaturated acid. Data in the same line of the same tissue with different superscripts are significantly different ($P < 0.05$).

Table S3

The content of FAAs in the muscle, hepatopancreas and ovaries of NFMC and BC (mg/g wet weight)

Amino acid	Muscle		Hepatopancreas		Ovaries	
	NFMC	BC	NFMC	BC	NFMC	BC
Asp	0.08 ± 0.01^{b}	0.06 ± 0.01^{a}	0.11 ± 0.02^{a}	0.09 ± 0.03^{a}	0.14 ± 0.00^{b}	0.09 ± 0.01^{a}
Thr	0.97 ± 0.08^{b}	0.35 ± 0.01^{a}	0.93 ± 0.27^{a}	1.65 ± 0.09^{b}	1.12 ± 0.06^{b}	0.79 ± 0.08^{a}
Ser	0.08 ± 0.01^{a}	0.09 ± 0.01^{a}	0.44 ± 0.11^{b}	0.19 ± 0.01^{a}	0.25 ± 0.01^{b}	0.13 ± 0.00^{a}
Glu	0.52 ± 0.03^{a}	0.69 ± 0.05^{a}	1.54 ± 0.31^{a}	1.86 ± 0.03^{a}	0.82 ± 0.04^{b}	0.57 ± 0.12^{a}
Gly	3.13 ± 0.06^{a}	3.17 ± 0.04^{a}	0.97 ± 0.22^{a}	1.79 ± 0.04^{a}	4.22 ± 0.05^{b}	3.24 ± 0.10^{a}
Ala	3.56 ± 0.08^{a}	3.63 ± 0.04^{a}	1.7 ± 0.41^{a}	2.15 ± 0.16^{a}	0.86 ± 0.16^{a}	1.11 ± 0.18^{a}
Cys	0.37 ± 0.01^{a}	0.38 ± 0.01^{a}	0.02 ± 0.00^{a}	0.17 ± 0.01^{b}	0.08 ± 0.01^{b}	0.04 ± 0.00^{a}
Val	0.42 ± 0.03^{b}	0.31 ± 0.01^{a}	2.18 ± 0.08^{b}	1.93 ± 0.09^{a}	1.98 ± 0.09^{b}	1.73 ± 0.09^{a}
Met	0.31 ± 0.02^{b}	0.21 ± 0.01^{a}	1.02 ± 0.03^{b}	0.62 ± 0.14^{a}	1.21 ± 0.01^{b}	0.98 ± 0.04^{a}
Ile	0.21 ± 0.02^{b}	0.06 ± 0.01^{a}	1.14 ± 0.08^{a}	0.82 ± 0.21^{a}	1.07 ± 0.06^{a}	1.14 ± 0.07^{a}
Leu	0.39 ± 0.04^{b}	0.28 ± 0.01^{a}	2.71 ± 0.15^{b}	2.19 ± 0.19^{a}	1.19 ± 0.02^{a}	2.02 ± 0.13^{a}
Tyr	0.15 ± 0.01^{b}	0.08 ± 0.01^{a}	1.49 ± 0.15^{b}	0.61 ± 0.12^{a}	1.66 ± 0.08^{a}	1.36 ± 0.14^{a}
Phe	0.45 ± 0.02^{b}	0.32 ± 0.01^{a}	1.66 ± 0.17^{a}	0.91 ± 0.21^{a}	1.25 ± 0.05^{a}	1.29 ± 0.11^{a}
Lys	0.24 ± 0.01^{a}	0.35 ± 0.01^{b}	0.41 ± 0.11^{a}	0.32 ± 0.01^{a}	0.33 ± 0.01^{b}	0.23 ± 0.03^{a}
His	0.34 ± 0.03^{a}	0.45 ± 0.01^{b}	0.45 ± 0.11^{a}	0.98 ± 0.06^{b}	0.46 ± 0.01^{a}	0.38 ± 0.09^{a}
Arg	1.56 ± 0.07^{a}	1.72 ± 0.02^{b}	0.39 ± 0.04^{b}	0.26 ± 0.03^{a}	0.53 ± 0.21^{a}	0.44 ± 0.04^{a}
Pro	2.45 ± 0.04^{a}	2.62 ± 0.03^{b}	3.96 ± 0.31^{a}	3.86 ± 0.16^{a}	1.31 ± 0.41^{a}	1.58 ± 0.13^{b}

Note: NFMC, normal female mud crab; BC, butter crab. Data are mean ± standard deviation (n = 4). Data in the same line of the same tissue with different superscripts are significantly different ($P < 0.05$).

Table S4

The content of free nucleotides in the muscle, hepatopancreas and ovaries of NFMC and BC (mg / 100 g)

Free Nucleotides	Muscle		Hepatopancreas		Gonads	
	NFMC	BC	NFMC	BC	NFMC	BC
CMP	0.81 ± 0.11[b]	0.00 ± 0.00[a]	1.50 ± 0.24[b]	0.21 ± 0.04[a]	25.97 ± 2.21[b]	15.31 ± 2.18[a]
AMP	70.25 ± 3.27[a]	143.54 ± 8.32[b]	9.21 ± 0.56[a]	17.98 ± 1.32[b]	45.26 ± 3.97[a]	48.38 ± 4.16[a]
UMP	1.49 ± 0.21[a]	1.51 ± 0.24[a]	21.32 ± 4.11[b]	5.22 ± 0.87[a]	85.11 ±5.12[b]	48.68 ±2.67[a]
GMP	2.87 ± 0.61[b]	0.53 ± 0.13[a]	12.86 ± 2.91[b]	5.78 ± 0.79[a]	120.32 ± 7.54[b]	86.56 ± 4.58[a]
IMP	4.36 ± 0.53[a]	7.27 ± 1.10[b]	0.11 ± 0.01[a]	9.56 ± 1.05[b]	0.22 ± 0.04[a]	0.95 ± 0.16[b]
Sum	79.77 ± 2.69[a]	152.85 ± 5.25[b]	45.00 ± 3.81[b]	38.75 ± 2.04[a]	276.88 ± 4.87[b]	199.88 ± 3.21[a]

Note: NFMC, normal female mud crab; BC, butter crab. Data are mean ± standard deviation (n = 4). Data in the same line of the same tissue with different superscripts are significantly different ($P < 0.05$).
